# Perfluoroalkyl and polyfluoroalkyl substances in consumer products

**DOI:** 10.1007/s11356-015-4202-7

**Published:** 2015-02-19

**Authors:** Matthias Kotthoff, Josef Müller, Heinrich Jürling, Martin Schlummer, Dominik Fiedler

**Affiliations:** 1 Fraunhofer Institute for Molecular Biology and Applied Ecology, Fraunhofer IME, Auf dem Aberg 1, 57392 Schmallenberg, Germany; 2 Fraunhofer Institute for Process Engineering and Packaging, Fraunhofer IVV, Giggenhauser Straße 35, 85354 Freising, Germany

**Keywords:** PFAA, PFAS, Perfluoroalkyl and polyfluoroalkyl chemicals, PFOS, PFOA, Consumer products, Food contact materials, Outdoor textiles

## Abstract

Perfluoroalkyl and polyfluoroalkyl substances (PFAS) are used in a wide range of products of all day life. Due to their toxicological potential, an emerging focus is directed towards their exposure to humans. This study investigated the PFAS load of consumer products in a broad perspective. Perfluoroalkyl sulfonic acids (C_4_, C_6_–C_8_, C_10_-PFSA), carboxylic acids (C_4_–C_14_-PFCA) and fluorotelomer alcohols (4:2, 6:2; 8:2 and 10:2 FTOH) were analysed in 115 random samples of consumer products including textiles (outdoor materials), carpets, cleaning and impregnating agents, leather samples, baking and sandwich papers, paper baking forms and ski waxes. PFCA and PFSA were analysed by HPLC-MS/MS, whereas FTOH were detected by GC/CI-MS. Consumer products such as cleaning agents or some baking and sandwich papers show low or negligible PFSA and PFCA contents. On the other hand, high PFAS levels were identified in ski waxes (up to about 2000 μg/kg PFOA), leather samples (up to about 200 μg/kg PFBA and 120 μg/kg PFBS), outdoor textiles (up to 19 μg/m^2^ PFOA) and some other baking papers (up to 15 μg/m^2^ PFOA). Moreover, some test samples like carpet and leather samples and outdoor materials exceeded the EU regulatory threshold value for PFOS (1 μg/m^2^). A diverse mixture of PFASs can be found in consumer products for all fields of daily use in varying concentrations. This study proves the importance of screening and monitoring of consumer products for PFAS loads and the necessity for an action to regulate the use of PFASs, especially PFOA, in consumer products.

**Electronic supplementary material:**

The online version of this article (doi:10.1007/s11356-015-4202-7) contains supplementary material, which is available to authorized users.

## Introduction

Perfluoroalkyl acids (PFAAs; nominations and abbreviations are kept according to Buck et al. 2011) have inimitable properties. Due to their amphiphilic properties, they are used for numerous technical applications, such as polymerisation aid, as industrial detergents, or in fire-fighting foams. They are also used as technical agents or raw materials for the production of water- and grease-repellent materials for all areas of use. However, they are resistant to biotic and abiotic breakdown; the resulting persistence of PFAAs is a problematic trait (Saez et al. 2008; Washington et al. 2010). So, a number of PFAAs can be found ubiquitously in the environment in Europe (Ahrens et al. 2011; Beskoski et al. 2013; Fliedner et al. 2012; Holmstrom and Berger 2008; Kwadijk et al. 2010; McLachlan et al. 2007; Ruedel et al. 2011; Verreault et al. 2007), northern America (Joyce Dinglasan-Panlilio et al. 2014), Asia (Kim et al. 2012; Takemine et al. 2014) and the oceans (Cai et al. 2012). Besides PFAA, a variety of polyfluoroalkyl substances occurs, but in contrast, these are biodegradable to a certain extent, instable in the environment and upon breakdown may form PFAA (Liu and Mejia Avendano 2013). Altogether, the mentioned chemicals are accounted as perfluoroalkyl and polyfluoroalkyl substances (PFAS). Findings of these substances in remote regions (Butt et al. 2007; Dietz et al. 2008; Reiner et al. 2011; Shi et al. 2010), in human blood (Brede et al. 2010; Eriksen et al. 2011; Holzer et al. 2011; Inoue et al. 2004; Karrman et al. 2007; Kato et al. 2011; Olsen et al. 2011), and breast milk (Antignac et al. 2013; Barbarossa et al. 2013; Fromme et al. 2010; Guerranti et al. 2013; Liu et al. 2010; Thomsen et al. 2010) are of very high concern, and publication numbers rise targeting these issues. The interest in human exposure to PFASs is accelerated by reports on their toxicological impacts in humans and animal models (Domingo 2012; Fabrega et al. 2014; Gascon et al. 2013; Hardell et al. 2014; Kjeldsen and Bonefeld-Jorgensen 2013; Lee and Viberg 2013; Peng et al. 2013; Rosen et al. 2013; Stein and Savitz 2011; Zhang et al. 2012).

Polyfluoroalkyl substances are surface-active moieties in polymeric surfactant materials and in polyfluoroalkyl phosphate surfactants (PAPS). Both are widely used in consumer products and industrial applications due to the inert and repellent characteristics they provide to surfaces they coat. Therefore, PFASs are used as detergents or impregnating agents in numerous industrial applications, such as paper and packaging (food and non-food applications) (Clara et al. 2008; Trier et al. 2011), as well as textile finishing (Herzke et al. 2012). The manufacture, use and disposal of consumer products are important sources for their emission into the environment, but the relative importance of direct and indirect sources vary for the different PFAA homologues and may be different on a local, regional or global scale (Armitage et al. 2009; Muller et al. 2012; Oliaei et al. 2013; Prevedouros et al. 2006; Wang et al. 2012).

In most cases, polyfluoroalkyl moieties originate from fluorotelomer alcohols (FTOH) as source, which are in turn major precursors for perfluorocarboxylic acid (PFCA) (D’Eon and Mabury 2011; Dinglasan et al. 2004; Ellis et al. 2004; Wallington et al. 2006). FTOH have been detected in ambient air and are sufficiently volatile to contribute to total human exposure with PFAS, as they may become part of the surrounding dust or degrade to PFAS before inhalation (Barber et al. 2007; Fraser et al. 2012, 2013; Haug et al. 2011; Huber et al. 2011; Jahnke et al. 2007; Langer et al. 2010; Schlummer et al. 2013).

Two major routes exist, by which polymeric surfactant materials and in PAPS may release monomeric PFASs to the environment: (i) non-polymerised remains are relieved from the material by means of wash out and/or evaporation, (ii) relieve upon breakage of covalent bonds integrating PFASs into polymer network.

The most frequently and most abundantly detected and investigated PFAS in almost any matrix is perfluorooctane sulfonic acid (PFOS), and often, perfluorooctanoic acid (PFOA) plays a major role. Both of these compounds are persistent, bio-accumulative and toxic (PBT) (ECHA 2011), and after PFOS, PFOA is officially classified as PBT in Europe; however, the labelling of this substance had so far not been harmonised on an EU level. Since June 27, 2008, the marketing and use of PFOS is prohibited with few exceptions in the EU (EU 2006, 2009, 2010). Maximum PFOS residue levels are set to 10.0 μg/kg when it occurs in substances or preparations and 1.0 μg/m^2^ in coated produce, such as textiles. PFOA is classified by the Risk Assessment Committee (RAC) of the Environmental Chemicals Agency (ECHA) as toxic after repeated exposure, carcinogenic and toxic to reproduction (ECHA 2011). RAC also agreed to classify the substance as acutely toxic by the oral and inhalation route, as severely damaging to the eye and as potentially harmful to breast-fed babies.

Available data on PFASs in consumer products are scarce to date. These data suggest extensive use in customer products, such as impregnating sprays, textiles, carpets, leather and cookware (Kissa 2001; Prevedouros et al. 2006). A study towards PFOA in some selected consumer products was conducted by Washburn et al. (2005). They found no significant linkage of PFOA loads in consumer products and human serum levels. Fraser et al. (2012, 2013) correlated the PFAS load of dust particles of indoor workplaces (i.e. offices, cars) with worker’s serum levels. They found offices to have the highest overall concentrations of PFASs; however, they concluded other than first suggested that ‘indoor dust may not be a significant source of exposure to PFAS for office workers’ (Fraser et al. 2012, 2013). Thus, other sources must apply at higher significances. However, a meta-analysis performed by Trudel et al. suggests a serious exposure to PFOS and PFOA by means of consumer products (Trudel et al. 2008). Most alarming at their findings is a predominant exposure of infants, toddlers and children due to their close contact to often highly loaded materials like carpets. An important issue in this regard are also precursors for PFCA, such as FTOH, as indirect sources. The goal of this study is the determination of PFASs in a large number of selected but versatile consumer products in order to get a comprehensive overview of the PFAS loads as potential sources for human exposure and intake pathways.

Therefore, analyses of PFAA as well as precursor substances like FTOH were performed in a versatile set of consumer contact and food contact materials. Perfluoroalkyl sulfonic (C_4_, C_6_–C_8_, C_10_-PFSA) and carboxylic acids (C_4_–C_14_-PFCA) as well as fluorotelomer alcohols (4:2, 6:2; 8:2 and 10:2 FTOH) were analysed in 115 samples of consumer products including textiles (outdoor materials), carpets, cleaning and impregnating agents, leather samples, baking and sandwich papers, paper baking forms and ski waxes. In order to allow for a large number of samples to be analysed, the parallel analysis for both, PFCA/PFSA and FTOH to allow a correlation of data was limited to some outdoor textiles. The gaseous emission of FTOH from some of these samples has been described elsewhere (Schlummer et al. 2013).

## Materials and methods

An experimental overview is only presented in brief, here (for further details see electronic Supplemental S1 and S2).

### Samples

The consumer product groups selected for investigation and the number of samples analysed are shown in Table 1. More details on the samples and the compounds analysed (perfluorocarboxylic and sulfonic acids and/or fluorotelomer alcohols) are given in Supplemental S1 (in the Electronic Supplementary Material).

**Table 1:** Consumer product groups and numbers of samples analysed

Product group	Samples analysed, *n* = 115	Analysed for PFSA and PFCA, *n* = 82	Analysed for FTOH, *n* = 36
Cleaning agents	9	6	3
Carpet samples	14	6	8
Impregnating sprays	16	3	13
Outdoor materials	5	3	4
Gloves	3	3	1
Leather samples	13	13	0
Individual paper-based FCM	33	33	0
Pooled paper-based FCM	7 (4–5 subsamples each)	0	7
Ski waxes	13	13	0
Wood glue	1	1	0
Awning cloth	1	1	0

The individual samples analysed were bought from local retailers or collected by co-workers of the institute or local clubs (e.g. ski waxes from local skiing club) in the first until third quarter of the year 2010. The sampled products span all quality levels from entry level to cutting edge products. The selection of the samples occurred randomly. The selection of samples was unbiased and reflects a possible real-life scenario of human contact to manifold products. To add to the data, next to the food contact papers bought within the indicated time frame, some older samples were collected from the staff of the involved institutes. The age of the samples ranged from few years to decades. These clearly biased samples are referred to as ‘archived samples’ and are used to estimate changes in PFAS loads after the EU-PFOS regulation.

### Target compounds (analytes)

The consumer products were analysed for 11 PFCAs (perfluorobutanoic acid (PFBA), perfluoropentanoic acid (PFPA), perfluorohexanoic acid (PFHxA), perfluoroheptanoic acid (PFHpA), perfluorooctanoic acid (PFOA), perfluorononanoic acid (PFNA), perfluorodecanoic acid (PFDA), perfluoroundecanoic acid (PFUnA ), perfluorododecanoic acid (PFDoA), perfluorotridecanoic acid (PFTrA), perfluorotetradecanoic acid (PFTeA), 5 PFSAs (perfluorobutanesulfonic acid (PFBS), perfluorohexanesulfonic acid (PFHxS), perfluoroheptanesulfonic acid (PFHpS), perfluorooctanesulfonic acid (PFOS), perfluorodecanesulfonic acid (PFDS), 4 fluorotelomer alcohols (4:2 FTOH, 6:2 FTOH, 8:2 FTOH, 10:2 FTOH) and perfluorooctane sulfonamide (PFOSA).

### Analysis

#### Standards

For most of the 17 PFAAs and all 4 analysed FTOHs, isotopically labelled standards were commercially available (Wellington Laboratories, bought from Campro Scientific) and applied as internal standards. The standards and their use for the quantitation of the individual analytes are listed in Supplemental S1 (PFAA) and S2 (FTOH) of the Electronic Supplementary Material.

#### Sample preparation

Depending on the matrix procedures using ion pair extraction, acidic-alkaline sequential extraction or solid-phase extraction (SPE) with weak anion exchangers were applied for sample preparation. The analytes were extracted with acetone or hexane resp. with methyl-t-butyl ether from alkaline environment in the presence of tetrabutylammonium hydrogensulfate (TBA) as ion pair reagent depending on the matrix. The quantitative determination was carried out by HPLC with mass spectrometric detection (HPLC-MS/MS). ^13^C-resp. ^18^O-labelled internal standards were used. For FTOH analysis, analytes were transferred into dichloromethane or hexane. Detailed information on the matrix specific extraction and sample preparation is provided in the Electronic Supplemental Material.

The methods applied differed in sample preparation and cleanup including the selection of solvents, depending on the respective matrix. The PFASs analysed represent the amounts of the compounds which are extractable by the used methods.

#### HPLC-tandem mass spectrometry of PFAA

The instrumental analysis of the PFAAs was carried out by HPLC-MS/MS in Fraunhofer IME while the fluorotelomer alcohols were analysed by GC/CI-MS in Fraunhofer IVV. Detailed information on the analytical methods used is given in Supplemental S1 (PFAA) and S2 (FTOH) of the Electronic Supplementary Material.

PFAA were separated by HPLC using a Waters Acquity system coupled to a Waters Tandem Quadrupole Detector (TQD) mass spectrometer (atmospheric pressure ionisation, negative ion electrospray mode). Generally, the HPLC-MS/MS procedure is considered to be a highly specific technique due to the tandem mass spectrometric detection. Chromatographic conditions, mass spectrometric parameters and mass transitions are provided in Supplemental S1 (PFAA) and S2 (FTOH) of the Electronic Supplementary Material. To be aware of the authenticity of PFBA, we used isotopically labelled PFBA as IS and applied a matrix spiked fortification reference. In case of doubt, PFBA was cross-checked with high-resolution MS/MS (Orbitrap LTQ Discovery XL) for the accurate mass for verification.

#### GC/CI-MS of FTOH

FTOHs were analysed by GC-MS with chemical ionisation (GC/CI-MS) using a HP 5890 Series II gaschromatograph coupled to a Finnigan MAT TSQ 7000 triple quadrupole mass spectrometer in PCI ionisation mode with methane 5.5 as reaction gas.

#### Quantification

Evaluation was made by internal standardisation using the mass-labelled internal standards shown in Table S2 (in the Electronic Supplementary Material). Calibration was performed using a minimum of six calibration solutions. Linear correlation coefficients of the calibration functions were typically >0.998. Reported concentrations are given as μg/kg and/or μg/m^2^, μg/kg is also used for liquid samples (i.e. cleaning agents) since this product group shows a very inhomogeneous consistency, ranging from liquids to foams.

#### Limits of quantification

For the PFAAs, limits of quantification (LOQs) of 0.1–0.5 μg/kg or 0.02–0.5 μg/m^2^ were derived from the extrapolation of the calibration lines according to the German standard DIN 32645. To simplify the evaluation for this study, LOQs were routinely set to 0.5 or 0.5 μg/m^2^ for all analytes in all matrices. This also stresses the focus of this study in dealing with highly contaminated samples relevant for exposure scenarios. For the FTOHs, LOQs were determined accordingly and vary from 0.3 to 0.8 μg/m^2^ for textiles, to 1.0 μg/kg for mixed paper samples. As cleaning and impregnating sprays (liquids) were diluted during sample pre-treatment, LODs of FTOH were significantly higher with up to 20,000 μg/kg. For statistical evaluation and visualisation of the data in diagrams, concentrations below the LOQ were considered as zero, and thus, the diagrams are scaled to 0.5 μg/kg or 0.5 μg/m^2^.

### Quality assurance

Both laboratories (at Fraunhofer IME and Fraunhofer IVV) hold accreditations according to DIN EN ISO/IEC 17025:2005 for the applied methods. Internal and external quality assurance measures for the analytical methods were applied accordingly. During each measurement series, quality control standards were analysed (about every 20th sample) to check the method performance. The absolute recoveries of the mass-labelled internal standards in the PFAA analysis ranged from 31 to 108 %. The recoveries of determinations were, with exceptions, in the range from 50 to 150 % which is in line with the requirements of DIN 38414, part 14 (S14) for PFAS determinations in sewage sludge, compost and soil.

In general, samples were extracted and analysed twice and the values were averaged. However, to estimate the precision of the chemical analysis, replicate measurements (2 to 12) of representative samples in each series were performed. For all analytes in all samples, 1280 data points were recorded; of these, 554 were above the LOQ. Thereof, 81 % have relative standard deviations (RSDs) below 40 % (69 < 30 %). All procedural and instrumental blanks were below the LOQ.

## Results

This study is a comprehensive approach analysing 115 samples of versatile consumer products for their load with perfluoroalkyl carboxylic (C_4_–C_14_-PFCA), perfluoroalkane sulfonic acids (C_4_, C_6_–C_8_, C_10_-PFSA) and fluorotelomer alcohols (4:2, 6:2; 8:2 and 10:2 FTOH).

Due to the high number of samples, a parallel analysis of all samples for all analytes was not aspired but performed for a limited set of textile samples. In order to reach a limited level of comparability, the investigated consumer products were grouped and displayed in graphs by means of statistical parameters (i.e. minima, maxima and median) of each group. The major groups that have been subject to analyses for all analyte groups are (i) cleaning agents, (ii) outdoor textiles, (iii) gloves, (iv) carpets, (v) impregnating and nanosprays, and (vi) paper-based food contact materials (FCM) such as baking papers and paper baking forms. PFAAs were additionally analysed in ski waxes, wood glues and awning textiles, as well as leather samples.

Results below the LOQ were stringently accounted as zero and used as a regular result for the determination of the median. An overview of all sample loads is provided in Table 2; the fraction of samples with no detectable PFAS is given in Table 3.

**Table 2:** Comprehension of measured PFAS concentrations by sample groups

	Cleaner		Wood glue		Nanosprays and impregnation sprays		Outdoor textiles		Carpet		Gloves		Paper-based FCM		Ski wax		Leather		Awning cloth	
	Max	Median	Max	Median	Max	Median	Max	Median	Max	Median	Max	Median	Max	Median	Max	Median	Max	Max	Max	Median
PFBA	-:-	-:-	-:-	-:-	2.5	1.4	6.1	0.5	14.7	-:-	1.2	0.8	9.9	0.7	362.1	14.3	241.8	241.8	0.5	0.5
PFPA	-:-	-:-	-:-	-:-	-:-	-:-	39.7	2.3	4.4	1.4	76.1	3.1	33.3	15.4	440.3	18.6	197.0	197.0	8.5	5.8
PFHxA	-:-	-:-	-:-	-:-	14.1	6.9	17.1	1.5	0.8	-:-	2.6	1.3	182.8	1.4	1737.1	17.9	4.5	4.5	1.0	0.9
PFHpA	-:-	-:-	-:-	-:-	4.2	2.7	4.6	-:-	1.4	-:-	1.3	0.6	379.3	-:-	424.8	7.6	1.6	1.6	-:-	-:-
PFOA	1.1	0.7	-:-	-:-	28.9	15.9	41.0	6.0	1.1	-:-	15.9	9.3	658.1	3.2	2033.1	15.5	12.4	12.4	10.9	5.8
PFNA	-:-	-:-	-:-	-:-	8.0	2.8	8.3	1.0	1.2	-:-	5.7	2.9	478.2	0.5	678.0	10.7	1.9	1.9	3.9	3.7
PFDA	-:-	-:-	-:-	-:-	10.7	8.1	12.6	2.6	1.0	-:-	10.2	5.7	489.4	2.5	1840.5	22.6	0.9	0.9	-:-	-:-
PFUnA	-:-	-:-	-:-	-:-	3.8	1.4	4.0	0.5	0.5	-:-	3.0	1.5	306.7	-:-	411.5	6.2	0.6	0.6	1.4	-:-
PFDoA	-:-	-:-	-:-	-:-	5.3	3.5	8.4	1.4	1.3	-:-	10.2	5.3	244.4	2.0	1441.9	16.1	3.6	3.6	1.4	1.4
PFTrA	-:-	-:-	-:-	-:-	1.2	0.5	1.5	-:-	-:-	-:-	4.5	2.0	36.6	-:-	179.4	4.0	0.9	0.9	0.8	0.8
PFTeA	0.8	-:-	-:-	-:-	1.9	1.1	8.8	0.7	1.1	-:-	24.8	10.1	22.1	1.3	745.2	9.8	1.4	1.4	0.9	0.8
PFBS	-:-	-:-	-:-	-:-	-:-	-:-	-:-	-:-	26.8	-:-	2.0	0.5	-:-	-:-	3.1	-:-	143	143	-:-	-:-
PFHxS	-:-	-:-	-:-	-:-	-:-	-:-	-:-	-:-	-:-	-:-	-:-	-:-	0.6	-:-	9.3	-:-	10.1	10.1	-:-	-:-
PFHpS	-:-	-:-	-:-	-:-	-:-	-:-	0.3	-:-	-:-	-:-	-:-	-:-	0.8	-:-	5.3	-:-	1.3	1.3	-:-	-:-
PFOS	1.6	1.2	-:-	-:-	-:-	-:-	35.4	9.5	2.9	1.0	11.9	1.5	8.8	0.7	159.8	1.6	5.6	5.6	2.3	1.1
PFDS	-:-	-:-	-:-	-:-	-:-	-:-	-:-	-:-	-:-	-:-	-:-	-:-	1.7	-:-	1.3	-:-	0.7	0.7	-:-	-:-
FTOH 4:2	-:-	-:-	~	~	329,000	-:-	-:-	-:-	-:-	-:-	-:-	-:-	-:-	-:-	~	~	~	~	~	~
FTOH 6:2	38,700	38,000	~	~	440,000	19,000	15.8	6.5	21.2	11.0	9.0	8.2	5.4	6.0	~	~	~	~	~	~
FTOH 8:2	547,100	63,000	~	~	719,300	146,200	379.9	44.2	32.8	17.2	58.6	53.2	14.1	15.7	~	~	~	~	~	~
FTOH 10:2	81,900	22,600	~	~	369,000	70,500	221.9	26.0	19.5	6.8	30.7	27.9	3.9	4.3	~	~	~	~	~	~

Data represent results in micrograms per kilogram (μg/kg), except for outdoor textiles, carpet, leather and awning cloth, which are in micrograms per square meter (μg/m^2^). For number of samples analysed, see Table 1

-:- below limit of quantification, ~ no samples were analysed

**Table 3:** Overview of the percentage of samples with detected PFAS

	Cleaner (%)	Wood glue (%)	Nanosprays and impregnation sprays (%)	Outdoor textiles (%)	Carpet (%)	Gloves (%)	Paper-based FCM (%)	Ski wax (%)	Leather (%)	Awning textiles (%)
PFBA	0	0	56	33	50	100	44	65	100	0
PFPA	0	0	22	78	90	0	73	100	13	0
PFHxA	0	0	67	56	40	100	27	88	54	0
PFHpA	0	0	33	33	15	100	23	69	92	0
PFOA	5	0	78	100	30	100	48	88	63	100
PFNA	0	0	56	67	20	100	24	73	92	100
PFDA	0	0	67	100	20	100	29	88	96	0
PFUnA	0	0	33	67	5	83	0	69	0	0
PFDoA	0	0	67	89	20	100	24	85	88	100
PFTrA	0	0	0	44	0	67	23	73	21	100
PFTeA	5	0	44	89	25	83	32	96	21	100
PFBS	0	0	0	0	45	0	0	38	100	100
PFHxS	0	0	0	0	0	0	6	35	96	0
PFHpS	0	0	0	0	0	0	2	27	100	100
PFOS	10	0	100	100	90	0	69	100	4	0
PFDS	0	0	0	0	0	0	6	15	79	0
PFOSA	n/d	n/d	n/d	n/d	0	n/d	0	8	46	100
4:2 FTOH	0	n/d	0	0	0	8	0	n/d	n/d	n/d
6:2 FTOH	100	n/d	75	100	100	77	71	n/d	n/d	n/d
8:2 FTOH	100	n/d	100	100	100	92	86	n/d	n/d	n/d
10:2 FTOH	100	n/d	100	100	88	92	86	n/d	n/d	n/d

As such, all samples > LOQ were considered

*n*/*d* no samples were analysed

### PFAA in consumer products

**Fig. 1:**
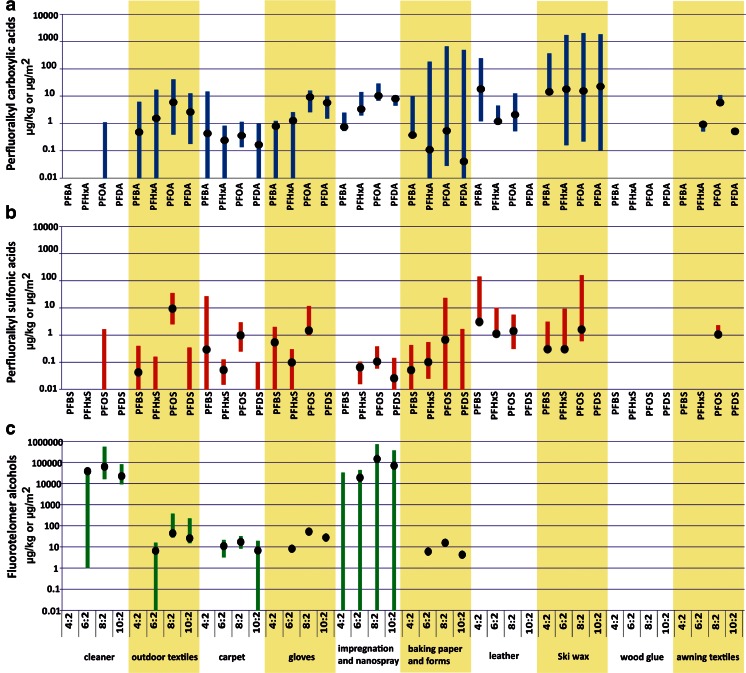
Shown are ranges of PFAS levels in selected consumer products. *Bars* reach from min to max values of analysis; *black dots* reflect the median. Units are μg/kg for cleaner, paper-based food contact materials (FCMs) and impregnating sprays, and μg/m^2^ for outdoor textiles, gloves and leather

The results for the major product groups are shown in Fig. 1a and b as concentrations (medians) and corresponding concentration ranges (min–max). A total of 82 samples were analysed for their load with PFAA.

The results show that there are consumer products with low or negligible PFSA and PFCA contents, such as the cleaning agents or the recent samples of baking and sandwich papers tested. On the other hand, high PFAS levels were identified in ski waxes, leather samples, outdoor textiles and some archived baking papers. Samples contaminated with significant levels of PFOA were found in all product groups except wood glue. Despite being in the legislative focus, PFOA and PFOS are the main contributors to the total PFAA loads.

The products with the highest PFCA and PFSA levels were ski waxes, with different PFCAs in concentrations in the milligram per kilogram (mg/kg) range and PFOS being the only sulfonic acid with considerable concentrations up to 159.8 μg/kg, followed by outdoor textiles, with values of 35.37 and 41.03 μg/kg for PFOS and PFOA, respectively. In the case of paper-based FCM, we analysed aged stock samples of muffin baking forms (before 2010, *n* = 3) and updated paper samples (from 2010, according to the actual sampling plan, *n* = 36). The high concentrations of PFCA found in paper-based FCM result from the few aged stock samples of muffin forms, which peak at 182.8, 658.1 and 489.4 μg/kg for PFHxA, PFOA and PFDA with their maximum values, respectively, and represent exceptions besides the updated commercial samples, where such peaks were not observed. However, across all analysed food contact paper samples (*n* = 39) in 66 % (*n* = 26) concentrations of ≥1 μg/kg of any PFCA/PFSA were detected. The most frequently and most abundantly detected substances are PFOS, PFBA, and importantly PFPA in all paper-based FCM, but in the three archived baking forms, PFOA is quantitatively the most abundant species with levels exceeding the concentrations of recent samples by two orders of magnitude. Notably, these samples had PFOS levels of only little above average concentration. The analytical results, given as maximal values (as a worst-case scenario) and respective median (as a realistic scenario) for all analyte groups, are gathered in Table 2. Transferring these result to mass by area, only the stock samples reach significant values of 15.1 mg/m^2^, the highest value for PFOS is 0.2 μg/m^2^ and thus below the EU regulation for coated produce. More detailed data on stored and recent paper-based FCM are provided in Supplemental S3 of the Electronic Supplemental Material.

### PFOS levels in consumer products with regard to European legislation

Moreover, the PFOS concentrations in carpet samples (up to 1.9 μg/m^2^), leather samples (up to 5 μg/m^2^) and outdoor materials (up to 10 μg/m^2^) exceeded the regulatory threshold value of 1 μg/m^2^ PFOS according to the European PFOS regulation (EU 2010).

Exceedance factors of this regulatory threshold for PFSA (and PFCA) in selected sample groups carpets, outdoor materials and leather samples are shown in Table 4. The exceedance factors for all analytes base on the EU regulation that limits PFOS at 1 μg/m^2^ in coated textiles and are adopted accordingly, also for non-regulated PFAS.

**Table 4:** Exceedance factors of PFASs for carpets, outdoor materials and leather samples, based on EU regulation for PFOS (1 μg/m^2^)

Substance	Carpets (*n* = 6)		Outdoor clothing (*n* = 6)		Leather (*n* = 13)	
	Median	Range	Median	Range	Median	Range
PFBS	15.1	9.2–19.5	<LOQ	<LOQ	4.4	0.8–120.1
PFOS	1.3	0.8–1.9	5.4	2.8–10.4	1.3	0.6–5.0
PFBA	7.5	3.5–12.1	<LOQ	<LOQ	18.8	1.4–227.9
PFPA	1.4	0.9–3.8	<LOQ	<LOQ	38.8	15.7–197.0
PFHxA	<LOQ	<LOQ	1.2	0.5–8.0	<LOQ	<LOQ
PFOA	0.8	<LOQ–0.8	4.1	0.9–19.0	2.2	0.8–11.2
Fraction of samples exceeding EU limit on PFOS	83 %		100 %		77 %	

Selected PFAS are given as examples

### FTOHs in consumer products

A total of 59 samples were analysed for their load with FTOHs. Of these, 29 samples were analysed individually and 30 paper-based FCM were equally pooled into mixed samples with four to five papers of common intended purpose, such as folding box boards, muffin and baking papers, and boxes of diverse sweets, cardboard boxes, cheese-wrapping papers, egg boxes and others. FTOH concentrations (medians) and their ranges were gathered for the main product groups cleaning agents, outdoor textiles, carpets, gloves, impregnating sprays and nanosprays, and paper-based FCM in Fig. 1c. The most abundantly detected FTOH is FTOH 4:2, with maxima of 547.1 μg/kg and 719.3 mg/kg in cleaners and impregnation and nanosprays, respectively. Also, in outdoor textiles, FTOH 8:2 reach maximum levels of 379.9 μg/m^2^. Notably, none of the textile samples was free of FTOH 6:2, 8:2 or 10:2, and only one and two carpet and paper-based FCM samples each were below LOQ of FTOH 8:2 and 10:2, respectively. Only FTOH 4:2 rarely occurs in the analysed sample set (in an impregnation spray), and only at comparably low levels (compare Table 2).

### Relation between PFCAs, PFSAs and FTOHs

**Fig. 2:**
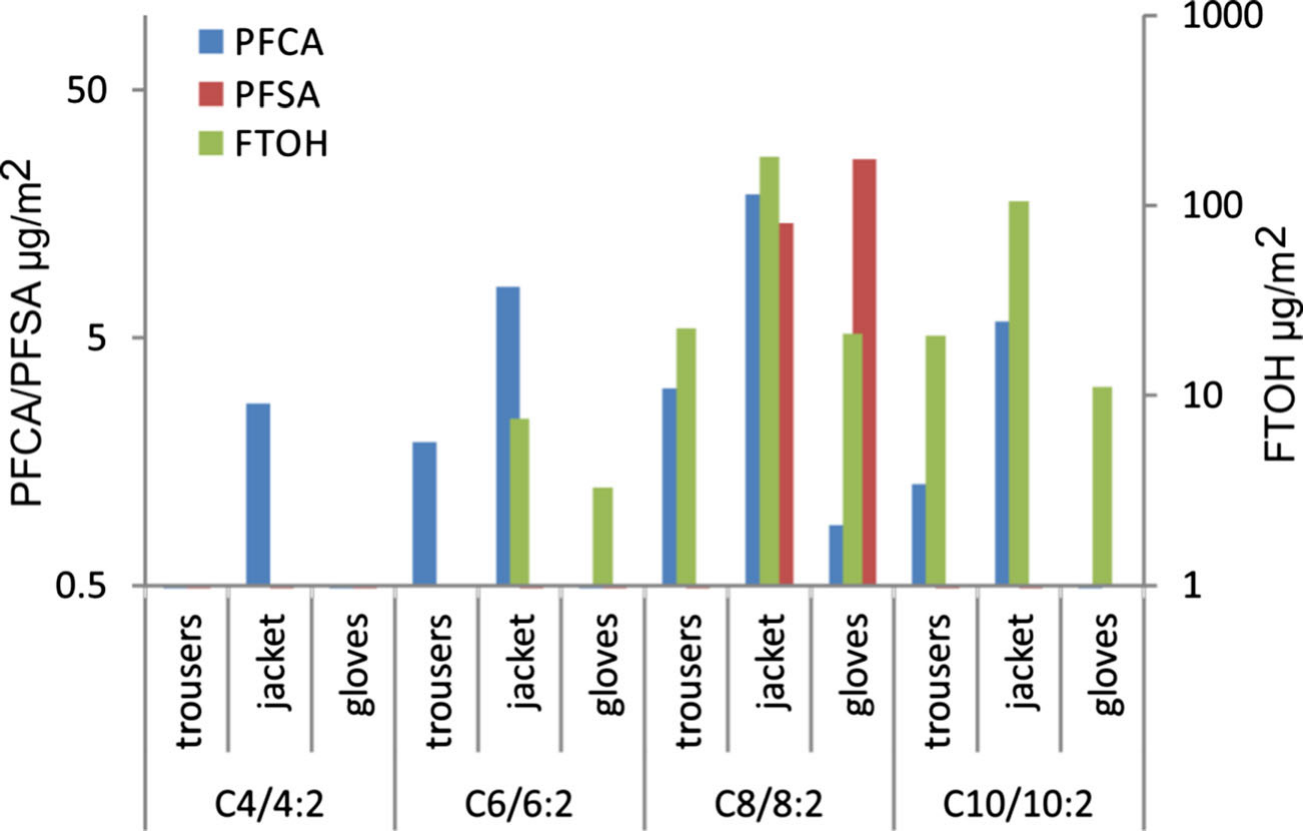
Bar chart of PFAS levels in three selected, parallel analysed textiles. Displayed are means of PFCA and PFSA of even chain lengths from C_4_ to C_10_ and their respective potential precursor FTOHs from 4:2 to 10:2

Concentration patterns given in Fig. 1a and c suggest a correlation between concentrations of FTOHs and PFCAs, at least for samples allowing for intra-sample comparisons. However, a parallel analysis of the same samples at both involved laboratories was limited to some outdoor textiles only (children’s outdoor trousers, an outdoor jacket and children’s gloves). PFAS levels of these samples were compared in Fig. 2. Here, 10:2 and 8:2 FTOH exceed levels of PFDA and PFOA by a factor of about 10. This is supported by a Pearson’s correlation analysis of FTOH and PFCA levels in outdoor textiles. FTOH 10:2 and 8:2 correlate with PFDA and PFOA with a correlation coefficient of *r* = 0.957 (*p* = 0.0013), whereas the correlation between FTOH on PFSA is significantly lower (*r* = 0.185; *p* = 0.3626).

## Discussion

### PFCA and PFSA in consumer products

PFASs are widely present in the investigated consumer products. The broad findings are summarised in Table 5. The only products with no or negligible PFAS concentrations tested were cleaners and wood glue. None of these samples exceeded an existing limit. In the majority of samples throughout all other sample groups, however, we found medium PFAS levels of up to 100 μg/kg or μg/m^2^ per substance. High concentrations over 100 μg/kg were reached in outdoor textiles, gloves, ski waxes and archived food contact paper samples. The degree of contamination does not seem to depend on quality level or price category of the tested products but was rather randomly distributed. Interestingly, a broad range of PFASs was found, rather than few distinctive substances, and does hence, not indicate a shift from the banned PFOS towards other PFASs conferring similar properties. Since there is no sample with direct use of PFOS, it is rather likely, that a contamination from unintentional production or cross-contamination occurred. The versatile pattern of PFCA with different chain lengths could rather indicate an unspecific formation route via harsh physical conditions like heat or UV exposure, i.e. hydrolysis and subsequent oxidation of FTOH from polymeric structures.

**Table 5:** Concluding overview of detected concentration ranges of PFCA/PFSA and FTOHs

No or negligible PFCAs/PFSAs	In the cleaning agents tested
Medium range concentrations, ≤100 μg/kg	FTOHs in cleaning agents and carpets
	Short-chain PFCAs/PFSAs in some carpets
	PFCAs in impregnating sprays
High concentrations, ≥100 μg/kg	FTOHs in impregnating sprays
	Outdoor materials and paper samples
	Short-chain PFCAs/PFSAs in some leather samples
	All PFCAs/PFSAs in some paper samples and some ski waxes
	Very high PFAA contents with values up to 2,000 μg/kg PFOA were detected in some ski waxes

In regard of customer safety, food contact materials play a crucial role as intake pathway, since hydrophobic contaminations, such as PFASs, are discussed to migrate into the fat- and protein-containing food matrix, especially in the presence of compounds like phospholipids or other surface active compounds (Prieto et al. 2004; Still et al. 2013; Trier et al. 2011). Other critical consumer products are any kind of impregnation or nanosprays, since during intended usage (the generation of aerosols), it can hardly be avoided to inhale at least some of the product. Clothing may, at least in part, play a role considering the skin as an intake route (compare Trudel et al.); moreover, for children’s clothing and in particular children’s gloves, the oral route becomes prominent in terms of a textile-to-mouth contact (Trudel et al. 2008). Similar considerations render carpets critical instances because of a hand-to-mouth route for children, especially toddlers and young children, as well as the formation and inhalation of dust from carpets for all customer ages. Nevertheless, many samples still do not match with existing PFOS regulation and exceed levels of 1 μg/m^2^ in coated materials or 10 μg/kg for substances and formulations, especially of the product groups outdoor textiles, food contact papers and carpets. Such contaminations are avoidable, if care is taken that not only the used raw materials (e.g. fabrics in the case of textiles) but also any associated materials and contact surfaces are free of it (e.g. in sewing plants), and therefore, the routes of the chemicals need to be traced to minimise the contact of consumers with PFOS and other PFASs. Moreover, this may indicate an exposure risk for staff within the value added chain where the contamination may occur.

The regulation of PFOS use may have caused shifts of the found PFAS spectra to alternative molecules. Albeit exhibiting different characteristics, in this study, the use was mainly shifted to, or remained at PFCA with shorter chain lengths in some carpets (loads >25 μg/m^2^ for PFBA); in the same samples, the PFOS load was inevitably lower (<2 μg/m^2^) compared to carpets with high PFOS loads. Next to this, we found odd-numbered substances like PFPA or PFNA to be major contributors in some samples, e.g. paper-based FCM and textiles (see Table 2).

The data do not allow for a detailed estimation of the sources for the versatile PFASs. In case of the textiles, it can be expected that the suppliers of raw materials avoid any contact at least to the banned PFOS. That we, nevertheless, found it in samples of the final textile products may be due to a contamination that occurred during the multi-step fabrication of the final products. An optimisation of the coating process, a monitoring of the raw materials and a critical check of all technical and accessory agents could reduce the contamination of consumer products with PFASs. In the past, PFOA was used as a process agent for fluoropolymers like PTFE and PVDF, and therefore, PFOA levels may be a result of using these polymers as a fibre material in our textiles. However, in the analysed textile samples, PFHxA and PFDA were found besides PFOA in concentrations up to 18.8 μg/kg. Both are chemicals, which exhibit similar characteristics compared to PFOA with chain lengths of −2 and +2, respectively. Their presence and comparable ratios of 10:2 and 6:2 FTOH, however, indicate that FTOH moieties in PAPS or polymeric surfactants may be the initial source of PFHxA, PFDA and probably PFOA.

By covalently attaching them to a fluorine-free polymer skeletal, FTOH are the monomeric basis for the final polymeric structures, e.g. on textiles and other consumer products. The found FTOH levels may thus reflect the success of binding reactions of monomers to the sole structures and thus represent technical process remnants (Dinglasan-Panlilio and Mabury 2006).

With respect to food contact paper baking forms, a temporal trend in terms of a ‘before and after 2010’ scenario was observed. The PFOS limit of 1.0 μg/m^2^ is exceeded only rarely and moderately for recently collected samples. In archived samples of the same product group, however, massive loads with perfluoroalkyl carboxylic acids were found. Such archived baking forms (purchased before 2010), e.g. popularly used for muffins, frequently show very high contaminations with PFASs (e.g. PFNA and PFDA up to 478.2 and 658.1 μg/kg, respectively). However, recently bought samples showed much lower values of max 13.5 and 18.0 μg/kg for PFOA and PFPA. Notably, in all recent food contact paper samples (purchased in 2010), PFPA was the main contributor to total PFAS load. In contrast to our findings, a recent study of food packaging papers from the Greek market found only a minor contamination of these products with selected PFASs (Zafeiraki et al. 2014); however, PFPA was not in their spectrum of monitored substances. Also, in other samples, PFPA was one of the most abundant and most frequently detected PFASs.

A dominant role of PFPA was not clear prior to this study, and PFPS was not part of the initially selected PFAS spectrum. Since PFBA and PFBS often occur with similar patterns, PFPS should be included in the substance spectra for future studies.

### FTOH in consumer products

The FTOH load of the investigated consumer products differed between product groups and inside the groups. In addition, considerable differences between the levels of PFAAs and FTOHs were observed. While the PFAA contents in the examined cleaning agents were negligible (<0.5 μg/kg, except for PFOS in one case (1.1 μg/kg)), the FTOH levels of cleaning agents were comparably high (up to 73,000 μg/kg 8:2 FTOH). In outdoor textiles, the FTOH levels topped 180 μg/m^2^ with PFOS levels of 10 μg/m^2^. This fact makes it hard to draw a general conclusion but requires exposure estimation from case to case.

In general, 6:2, 8:2 and 10:2 FTOH were identified in FTOH positive samples with 8:2 being the dominating congener. Only in mixed paper samples different patterns were observed, possibly due to the application of longer chain FTOH side groups in the specific surfactants used.

FTOH loads within the product groups were not distributed homogeneously, i.e. the concentrations differed from one sample to another. In the case of outdoor textiles, FTOH levels varied by a factor of about 25.

Highest FTOH levels were found in impregnating sprays (up to 719,000 μg/kg 8:2 FTOH), which is consistent with the assumption that polymeric surfactants used in impregnating applications are a major source of FTOH (Dinglasan-Panlilio and Mabury 2006). Thus, not using such sprays in closed rooms is a major and serious task to prevent direct customer exposure.

Outdoor textiles are another product group exhibiting high levels of FTOH (up to 380 μg/m^2^ 8:2 FTOH). These have been identified as major sources of FTOH in indoor environments as FTOH are released from these products (Schlummer et al. 2013; Langer et al. 2010).

Initial studies (Dinglasan-Panlilio and Mabury 2006) identified the presence of residual unbound FTOHs of varying chain lengths (C_6_–C_14_) in several commercially available and industrially applied polymeric and surfactant materials, normally used for impregnating of leather surfaces, papers or other materials. The authors concluded ‘that residual alcohols, left unreacted and unbound from the manufacturing process of fluorinated polymers and surfactants, could be a significant source of the polyfluorinated telomer alcohols and sulfonamides released into the environment’. The examined fluorinated materials contained 0.04–3.8 % residual-free fluorinated alcohols on an applied fluorinated alcohol basis (dry mass basis). This study suggests that elimination or reduction of these residual alcohols from all marketed fluorinated polymers and fluorosurfactants is the key in reducing the prevalence of perfluoroalkyl acids formed in the environment.

Ski waxes showed nearly the complete PFAS spectrum, and other samples were dominated by individual compounds. In case of FTOH, normally 6:2, 8:2 and 10:2 FTOH were identified with 8:2 being the dominating congener. Accordingly, Plassmann and Berger reported the finding of several PFAS in snow and soil samples from ski areas (Plassmann and Berger 2013). Adding to this context, a massive PFAS exposure of people professionally working with ski waxes has been reported (Nilsson et al. 2010). Moreover, degradation of FTOH to PFCA in humans was also shown using ski waxers by Nilsson et al., indicating the importance of such studies (Nilsson et al. 2013).

Only in mixed paper samples different patterns were observed, possibly due to the application of longer chain FTOH side groups in the specific surfactants used.

Regarding the co-occurrence of different PFAS subgroups, a significant linkage for FTOH and PFCA, but not for FTOH and PFSA was shown. The load with PFCAs could yet be depending on the post-coating processes of the textiles such as poor polymerisation conditions, insufficient linkage to the basic textile polymeric material (e.g. polyester), or the use of raw materials of suboptimal quality. As technically expected, due to different industrial synthesis routes and no degradation to sulfonic acids, the load with PFOS shows no correlation to the respective loads with FTOH.

### Comparison with literature data

In a similar approach, Herzke et al. (2012) analysed 30 consumer products from the Norwegian market and found beneath PFOS and PFBA shorter chained PFAS, such as PFBS, to be a major contributor to the total PFAS load of consumer products (especially in non-stick ware and waterproofing agents, but not in food contact cardboards) (Herzke et al. 2012). On average, they found less individual PFAS compared to our results and comparably high loads only in leather. However, the sample size of two per matrix was relatively small and due to the inhomogeneity of PFAS contamination, highly loaded samples were rarely found in this study, e.g. one aqueous fire-fighting foam and one waterproofing agent had extraordinary PFAS loads of up to 900 mg/L (1286 mg/kg; with an estimated density of 0.7 kg/L) PFHpS and 1.2 mg/L (1.7 μg/kg) PFDoA, respectively. Fire-fighting foams were not subject of our analysis, but in all analysed waterproofing agents (in our study, nanosprays and impregnating sprays), the highest PFCA load was found for PFOA with max 28.9 μg/kg, and max 5.3 μg/kg PFDoA, which fits with the results of Herzke et al., for the majority of samples analysed. Also, for FTOH, the results were comparable, with the exceptions given above. FTOH were also the majorly investigated substance group of a study by Fiedler et al. (2010). Also, here, in 15 samples analysed, among them 9 impregnating agents, the highest values were found for FTOH 8:2 and 10:2 with 52,000 and 32,000 μg/L in impregnating agents, which is in the same range as in this study. PFOS was not detected in any sample, but also, PFOA was found in levels of 100–400 μg/L. High values for PFAS in ski wax were also found by Freberg et al. (2010). Most alarming, corresponding PFAS levels were also found in the blood of customers having professional contact with these products (Freberg et al. 2010; Nilsson et al. 2010).

The widespread use of PFASs in consumer-near products is reported by Huset et al. (2011) who analysed municipal landfill leachates. The authors found PFCA and PFSA levels of up to 2.8 and 2.3 μg/L, respectively, while short-chain PFASs (C_4_–C_7_) were more abundant than the corresponding longer chain homologues. Results of our study underline the dominance of short chain PFAS in two product categories of consumer products with significant levels of perfluoroalkyl compounds: leather and carpets and particularly PFPA in paper-based FCM.

A comprehensive overview on PFAS in outdoor textiles is provided in a non-peer-reviewed publication by the interest group Greenpeace e.V. organisation. In this large-scale approach, conditionally, they found a variety of PFAS in qualitatively and quantitatively comparable concentrations to the results of our study (Santen and Kallee 2012). However, they reported no textile exceeding the EU regulation for PFOS. This may be due to the selection of samples, and a role may also play the sampling years for our (2010) and the Greenpeace study (2012). Berger and Herzke (2006) reported high amounts of extractable FTOH (sum of 4:2 to 10:2) in different textiles samples including outdoor textiles. Median levels of their report compare well to FTOH levels reported here.

### Implication for risk assessment

Exposure estimations can be optimised when detailed information is available for as many as possible samples. Thus, this and further data can contribute to reliable safety considerations (e.g. Trudel et al. 2008) and point out the importance of a large-scale monitoring approach for PFASs in a broad spectrum of consumer products and food contact materials. An important issue for the risk assessment, however, is the risk of cumulative exposure of all PFASs in sum (Borg et al. 2013), e.g. the 16 monitored PFCA and PFSA sum up to 113.7 μg/kg in children’s gloves, with PFPA being the main contributor at a concentration of 47.7 μg/kg. Borg et al. monitored the levels in blood of respective customer groups, and our data provide information on contact points. In either case (Borg et al. analysed 17 substances), considering only selected compounds for exposure assessment may lead to an underestimation of the risk. Future approaches should therefore target an enhancement of analyte portfolio towards further polyfluoroalkyl and branched substances in order to allow for comprehensive exposure estimation.

Generally, the PFAS exposure of consumers by products like outdoor materials (textiles) needs to be better understood. Markedly, also children’s textiles and carpets are highly loaded with diffuse mixes of PFAS and may contribute to the exposure of children and toddlers. According to the tolerable daily intake (TDI) defined for PFOA (1.5 μg/kg body weight/day), the maximum exposure for a toddler of 10 kg is 15 μg per day (EFSA 2008). With up to 41.0 μg/m^2^ in outdoor textiles, a significant portion of the TDI may be contributed by these products upon textile-mouth or hand-mouth contact. Up to now, it can only be estimated that these limits are not passed from a toddler wearing gloves via the hand to mouth route for a single compound or even in a cumulative exposure scenario (Trudel et al. 2008). However, these considerations need to be subject of future research.

This study confirmed the presence of PFASs in a wide variety of consumer products including sensitive samples such as children’s clothing. Moreover, many products were identified which do not comply with the present European PFOS regulation. Other samples, such as food contact papers, impregnation sprays and ski waxes showed massive loads with PFCA instead PFSA.

## Electronic supplementary material

Below is the link to the electronic supplementary material.

ESM 1 (DOCX 96 kb)
