# Supplementary material for: Perfluoroalkyl and polyfluoroalkyl substances in consumer products
Source: Environ Sci Pollut Res Int. 2015 Feb 19;22(19):14546–59. doi: 10.1007/s11356-015-4202-7 (PMC4592498; doi:10.1007/s11356-015-4202-7)
Supplement: Supplementary file 1 — (DOCX 96 kb) [file 11356_2015_4202_MOESM1_ESM.docx]

Electronic Supplementary Material

Journal: Environmental Science and Pollution Research’

Title: Per- and Polyfluoroalkyl Substances in Consumer Products

Authors: Matthias Kotthoff, Josef Müller, Heinrich Jürling, Martin Schlummer,
Dominik Fiedler

Corresponding author:
Dr. Matthias Kotthoff
Fraunhofer Institute for Molecular Biology and Applied Ecology (Fraunhofer IME), Auf dem Aberg 1
57392 Schmallenberg, Germany
Email: matthias.kotthoff@ime.fraunhofer.de
Phone: +49 2972 302 472
Fax: +49 2972 302 319

Inhalt

[1. Supplemental S1: Analytical methods for the determination of perfluoroalkyl carboxylic and perfluoroalkane sulfonic acids (PFAAs) in consumer products 4](#_Toc379362745)

[1.1 Scope 4](#_Toc379362746)

[1.2 Method summary 5](#_Toc379362747)

[1.3 Materials 6](#_Toc379362748)

[1.3.1 Reagents 6](#_Toc379362749)

[1.4 Solutions 6](#_Toc379362750)

[1.5 Performance 7](#_Toc379362751)

[1.5.1 Sample preparation 7](#_Toc379362752)

[1.5.2 Analysis of cleaning agents 7](#_Toc379362753)

[1.5.3 Analysis of carpets 7](#_Toc379362754)

[1.5.4 Analysis of impregnating sprays 7](#_Toc379362755)

[1.5.5 Analysis of outdoor materials 8](#_Toc379362756)

[1.5.6 Analysis of leather samples 8](#_Toc379362757)

[1.5.7 Analysis of paper based food contact materials 8](#_Toc379362758)

[1.5.8 Analysis of ski waxes 8](#_Toc379362759)

[1.5.9 Analysis of wood glue and awning cloth 9](#_Toc379362760)

[1.6 Instrumental analysis 10](#_Toc379362761)

[1.6.1 HPLC parameters 10](#_Toc379362762)

[1.6.2 MS-MS parameters 10](#_Toc379362763)

[1.7 Identification 11](#_Toc379362764)

[1.8 Evaluation 11](#_Toc379362765)

[2. Supplemental S2: Analytical methods for the determination of fluorotelomer alcohols (FTOHs) in air samples, impregnating sprays and solid samples 13](#_Toc379362766)

[2.1 Scope 13](#_Toc379362767)

[2.2 Method summary 14](#_Toc379362768)

[2.3 Equipment 14](#_Toc379362769)

[2.4 Materials 15](#_Toc379362770)

[2.4.1 Reagents 15](#_Toc379362771)

[2.4.2 Solutions 15](#_Toc379362772)

[2.5 Performance 15](#_Toc379362773)

[2.5.1 Sample preparation 15](#_Toc379362774)

[1.1.1 Air samples 16](#_Toc379362775)

[1.1.2 Emissions of consumer products 17](#_Toc379362776)

[1.1.3 Liquid samples 17](#_Toc379362777)

[1.1.4 Solid samples 17](#_Toc379362778)

[2.6 Instrumental parameters 17](#_Toc379362779)

[2.6.1 GC parameters (example) 17](#_Toc379362780)

[2.6.2 MS-MS parameters (example) 17](#_Toc379362781)

[2.7 Data acquisition 19](#_Toc379362782)

[2.8 Identification 19](#_Toc379362783)

[2.9 Evaluation 19](#_Toc379362784)

[2.10 Control standards and blanks 19](#_Toc379362785)

[Supplemental S3: PFAA contents of stored and recent paper based food contact materials. 20](#_Toc379362786)

# Supplemental S1: Analytical methods for the determination of perfluoroalkyl carboxylic and perfluoroalkane sulfonic acids (PFAAs) in consumer products

## Scope

The following analytical method describes the quantitative determination of 17 perfluorinated compounds (see Table 1) in consumer products. Quantification is carried out by the use of native standards and nine ^13^C- resp. ^18^O-labelled internal standards (IS).

Table 1: Perfluoroalkyl carboxylic and perfluoroalkane sulfonic acids

| **Acronym Name CAS** | **number* Molecular** | **formula** |  |
| --- | --- | --- | --- |
| **Target** | **analytes** |  |  |
| PFBA | Perfluorobutanoic acid | 375-22-4 | C_3_F_7_COOH |
| PFPA | Perfluoropentanoic acid | 2706-90-3 | C_4_F_9_COOH |
| PFHxA | Perfluorohexanoic acid | 307-24-4 | C_5_F_11_COOH |
| PFHpA | Perfluoroheptanoic acid | 375-85-9 | C_6_F_13_COOH |
| PFOA | Perfluorooctanoic acid | 335-67-1 | C_7_F_15_COOH |
| PFNA | Perfluorononanoic acid | 375-95-1 | C_8_F_17_COOH |
| PFDA | Perfluorodecanoic acid | 335-76-2 | C_9_F_19_COOH |
| PFUnA | Perfluoroundecanoic acid | 4234-23-5 | C_10_F_21_COOH |
| PFDoA | Perfluorododecanoic acid | 307-55-1 | C_11_F_23_COOH |
| PFTrA | Perfluorotridecanoic acid | 72629-94-8 | C_12_F_25_COOH |
| PFTeA | Perfluorotetradecanoic acid | 376-06-7 | C_13_F_27_COOH |
| PFBS | Perfluorobutanesulfonic acid | 59933-66-3 | C_4_F_9_SO_3_H |
| PFHxS | Perfluorohexanesulfonic acid | 355-46-4 | C_6_F_13_SO_3_H |
| PFHpS | Perfluoroheptanesulfonic acid | 375-92-8 | C7F15SO3H |
| PFOS | Perfluorooctanesulfonic acid | 1763-23-1 | C_8_F_17_SO_3_H |
| PFDS | Perfluorodecanesulfonic acid | 335-77-3 | C_10_F_21_SO_3_H |
| PFOSA | Perfluorooctanesulfon amide | 754-91-6 | C_8_F_17_SO_2_NH_2_ |

Table 2: List of Internal standards (IS)

| MPFBA | Perfluoro-n-[1,2,3,4-^13^C_4_]butanoic acid | n.a. | ^13^C_3_F_7_^13^COOH |
| --- | --- | --- | --- |
| MPFHxA | Perfluoro-n-[1,2-^13^C_2_]hexanoic acid | n.a. | ^13^C_2_^12^C_3_F_11_COOH |
| MPFOA | Perfluoro-n-[1,2,3,4-^13^C_4_]octanoic acid | n.a. | ^13^C_4_^12^C_3_F_15_COOH |
| MPFNA | Perfluoro-n-[1,2,3,4,5-^13^C_5_]nonanoic acid | n.a. | ^13^C_5_^12^C_3_F_17_COOH |
| MPFDA | Perfluoro-n-[1,2-^13^C_2_]decanoic acid | n.a. | ^13^C_2_^12^C_7_F_19_COOH |
| MPFUnA | Perfluoro-n-[1,2-^13^C_2_]undecanoic acid | n.a. | ^13^C_2_^12^C_10_F_21_COOH |
| MPFDoA | Perfluoro-n-[1,2-^13^C_2_]dodecanoic acid | n.a. | ^13^C_2_^12^C_11_F_23_COOH |
| MPFHxS | Perfluoro-n-[^18^O_2_]hexanesulfonic acid | n.a. | C_6_F_13_S^18^O_2_^16^OH |
| MPFOS | Perfluoro-n-[1,2,3,4-^13^C_4_]octanesulfonic acid | n.a. | ^13^C_4_^12^C_4_F_17_SO_3_H |

*Salts of the analytes have different CAS numbers, n.a. not available

The native PFAS were manufactured by Wellington Laboratories and bought from Campro Scientific as certified mixtures:

Linear Perfluoroalkyl carboxylic acids

**Name Lot number purity* concentration acid***

PFC-MXA PFCMXA0507 ≥ 98 % 2.00

Linear Perfluoroalkane sulfonic acids

**Name Lot number purity concentration salt* concentration anion****

PFC-MXA PFSMXA0607 ≥ 98 % 2.00 µg/mL 1.77 – 1.93 µg/mL

* per compound
** depending on respective substance

PFOSA (100 %, Apollo) and the mass labeled internal standards (solutions in methanol, 50 µg/mL, Wellington Laboratories resp. Campro Scientific) were used as single substances.

Table 3 Purity of the used internal standards

|  | **Chemical purity** | **Isotopic purity** |
| --- | --- | --- |
| MPFBA | > 98 % | ≥ 99 % ^13^C |
| MPFHxA | > 98 % | ≥ 99 % ^13^C, C_1_ – C_2_ |
| MPFOA | > 98 % | ≥ 99 % ^13^C, C_1_ – C_4_ |
| MPFNA | > 98 % | ≥ 99 % ^13^C, C_1_ – C_5_ |
| MPFDA | > 98 % | ≥ 99 % ^13^C, C_1_ – C_2_ |
| MPFUnA | > 98 % | ≥ 99 % ^13^C, C_1_ – C_2_ |
| MPFDoA | > 98 % | ≥ 99 % ^13^C, C_1_ – C_2_ |
| MPFHxS | > 98 % | > 94 % ^18^O_2_ |
| MPFOS | > 98 % | > 99 % ^13^C, C_1_ – C_4_ |

## Method summary

The analytes shown in Table 1 were extracted with acetone or hexane resp. with methyl-t-butylether from alkaline environment in the presence of tetrabutylammonium hydrogensulfate (TBA) as ion pair reagent depending on the matrix. The procedures are given below. The quantitative determination was carried out by HPLC with mass spectrometric detection (HPLC-MS-MS). ^13^C- resp. ^18^O-labelled internal standards were used.

## Materials

### Reagents

Table 4: The following chemicals or reagents are examples, other brands of corresponding quality can be used.

| Used chemical | purity |  |
| --- | --- | --- |
| water for HPLC | >18,2 MΩ | e.g. Millipore Alpha Q |
| methanol | 99.8 % | HPLC-grade, Mallinckrodt Baker |
| hexane | 99.0 % | nanograde, Mallinckroth |
| acetone | 99 %, p.a. | e.g. Merck, for analysis |
| ammonium acetate | 98 %, p.a. | Fluka/Sigma Aldrich |
| ammoniumhydroxide | 25 %, p.a. | Merck |
| methyl-tert.-butylether (MTBE) | 99.7 % | Pestanal, Riedel de Haen |
| tetrabutylammonium hydrogensulfate | > 99 % | pur., Fluka |
| Na_2_CO_3_ und NaHCO_3_ | 99.5 % | z. A., Merck |
| NaOH | 98-100,5 % | food grade, Merck |
| HCl | 99.9 % | z. A., Merck |

## Solutions

Internal standard solution

100 µL of each certified internal standard are transferred into a 50 mL measuring bulb and made up with methanol-water (50+50, v/v). The concentration is 100 ng/mL per standard.

Dilutions

The solutions of the analytes are diluted with methanol-water to concentrations of 0.24 µg/mL.

For the calibration further 8 - 10 dilutions are prepared in the range from 0.12 to 12 ng/mL. Internal standards are added to final concentrations of 10 ng/mL, each.

Control standards

Control standard solutions with concentrations of 2.4 ng/mL and 8.4 ng/mL were prepared. The control standard solutions are prepared independently from the calibration solutions.

0.5 M tetrabutylammonium hydrogensulfate (TBA) solution

169 g TBA are weighed accurately into a 1 L bulb. 500 mL of water is added and the pH adjusted to pH 10 by slowly adding of 44 mL to 54 mL of 10 N NaOH. After the pH is adjusted accurately the bulb is made up to 1 L with water. Then the TBA solution is transferred into a polypropylene bottle. The pH of 10 is checked daily and adjusted if necessary.

Na_2_CO_3_/ NaHCO_3_ solution

26.5 g Na_2_CO_3_ and 21 g NaHCO_3_ are weighed accurately and transferred into a 1 L bulb. The bulb is filled up to the mark with water and transferred into a polypropylene bottle. The resulting concentration is 0.25 N.

## Performance

### Sample preparation

The PFCs analysed represent the amounts of the compounds which are extractable by the used methods.

### Analysis of cleaning agents

Spray samples: Maximal 1 g of the sample is sprayed into a 15 mL polypropylen (PP) centrifuge tube. Liquid samples: 1 g of the sample is transferred into a 15 mL polypropylen (PP) centrifuge tube.

Then 100 µL of internal standard solution (9 internal standards, 100 ng/mL each) and 2 mL of 0.25 M Na_2_CO_3_ / NaHCO_3_ buffer, 1 mL of 0.5 M tetrabutylammonium hydrogensulfate (TBA) and 5 mL of methyl-t-butyl-ether (MTBE) is added (for the preparation of the Na_2_CO_3_ / NaHCO_3_ buffer and the TBA solution see Annex 1).

After vortexing for 60 min the mixture is centrifuged at 4000 rpm for 10 min. The clear supernatant is transferred into a further 15 mL PP tube. The solvent is evaporated by a stream of nitrogen to dryness. The residue is solved in 1 mL of methanol-water (50+50, v/v, 5 min ultrasonic bath) and the liquid filtered using a 0.45 µm RC Filter. The clear filtrate is transferred into an autosampler vial and measured by HPLC-MS-MS.

PFOSA could not be determined by this method, as fortification tests resulted in recoveries of < 10 %.

### Analysis of carpets

The samples are stamped out of the carpet using a hole punch (d=25 mm). Two stamps are used for one analytical sample und put into a 15 mL polypropylen (PP) centrifuge tube. Then 100 µL of internal standard solution (9 internal standards, 100 ng/mL each) and 10 mL of acetone is added. After 30 min of ultrasonic treatment the mixture is vortexed for about 30 min. Then the extract is transferred into a further 15 mL PP tube and the solvent evaporated by a stream of nitrogen to dryness. The residue is solved in 1 mL of methanol-water (50+50, v/v, 5 min ultrasonic bath) and the liquid filtered using a 0.45 µm RC Filter. The clear filtrate is transferred into an autosampler vial and measured by HPLC-MS-MS.

### Analysis of impregnating sprays

Maximal 1 g of sample is sprayed into a 15 mL polypropylen (PP) centrifuge tube. Then 100 µL of internal standard solution (9 internal standards, 100 ng/mL each) and 2 mL of 0.25 M Na_2_CO_3_ / NaHCO_3_ buffer, 1 mL of 0.5 M tetrabutylammonium hydrogensulfate (TBA) and 5 mL of methyl-t-butyl-ether (MTBE) is added (for the preparation of the Na_2_CO_3_ / NaHCO_3_ buffer and the TBA solution see Annex 1).

After vortexing for 60 min the mixture is centrifuged at 4000 rpm for 10 min. The clear supernatant is transferred into a further 15 mL PP tube. The solvent is evaporated by a stream of nitrogen to dryness. The residue is solved in 1 mL of methanol-water (50+50, v/v, 5 min ultrasonic bath) and the liquid filtered using a 0.45 µm RC Filter. The clear filtrate is transferred into an autosampler vial and measured by HPLC-MS-MS.

### Analysis of outdoor materials

4 cm^2^ of the outdoor materials were put into a 15 mL polypropylen (PP) centrifuge tube. Then 100 µL of internal standard solution (9 internal standards, 100 ng/mL each) and 2 mL of 0.25 M Na_2_CO_3_ / NaHCO_3_ buffer, 1 mL of 0.5 M tetrabutylammonium hydrogensulfate (TBA) and 5 mL of methyl-t-butyl-ether (MTBE) is added (for the preparation of the Na_2_CO_3_ / NaHCO_3_ buffer and the TBA solution see Annex 1).

After vortexing for 60 min the mixture is centrifuged at 4000 rpm for 10 min. The clear supernatant is transferred into a further 15 mL PP tube. The solvent is evaporated by a stream of nitrogen to dryness. The residue is solved in 1 mL of methanol-water (50+50, v/v, 5 min ultrasonic bath) and the liquid filtered using a 0.45 µm RC Filter. The clear filtrate is transferred into an autosampler vial and measured by HPLC-MS-MS.

PFOSA could not be determined by this method, as fortification tests resulted in recoveries < 10 %.

### Analysis of leather samples

The analytical sample is stamped out of the leather using a hole punch (d=25 mm) und put into a 15 mL polypropylen (PP) centrifuge tube. Then 100 µL of internal standard solution (9 internal standards, 100 ng/mL each) and 10 mL of acetone is added. After 30 min of ultrasonic treatment the mixture is vortexed for about 30 min. Then the extract is transferred into a further 15 mL PP tube and the solvent evaporated by a stream of nitrogen to dryness. The residue is solved in 1 mL of methanol-water (50+50, v/v, 5 min ultrasonic bath) and the liquid filtered using a 0.45 µm RC Filter. The clear filtrate is transferred into an autosampler vial and measured by HPLC-MS-MS.

### Analysis of paper based food contact materials

100 cm^2^ baking paper resp. 1 baking form is chopped and put into a 15 mL poly­propylen (PP) centrifuge tube. Then 100 µL of internal standard solution (9 internal standards, 100 ng/mL each) and 10 - 15 mL of acetone is added. After 10 min of ultrasonic treatment the mixture is vortexed for about 60 min. Then the extract is transferred into a further 15 mL PP tube and the solvent evaporated by a stream of nitrogen to dryness. The residue is solved in 1 mL of methanol-water (50+50, v/v, 5 min ultrasonic bath) and the liquid filtered using a 0.45 µm RC Filter. The clear filtrate is transferred into an autosampler vial and measured by HPLC-MS-MS.

### Analysis of ski waxes

About 0.1 to 0.3 g of the ski wax is weighed accurately and transferred into a 15 mL polypropylen (PP) centrifuge tube. Then 100 µL of internal standard solution (9 internal standards, 100 ng/mL each) and 5 mL of hexane is added. After vortexing for 30 min 1 mL of methanol is added and the vortexing continued for further 30 min. Then the mixture is centrifuged at 4000 rpm at 10°C for 10 min and the lower methanolic phase is transferred into a further 15 mL PP tube. The solvent is evaporated by a stream of nitrogen to dryness. The residue is solved in 1 mL of methanol-water (50+50, v/v, 5 min ultrasonic bath) and the liquid filtered using a 0.45 µm RC Filter. The clear filtrate is transferred into an autosampler vial and measured by HPLC-MS-MS.

### Analysis of wood glue and awning cloth

The wood glue and the awning cloth were analysed as the outdoor materials. About 0.5 g of the glue and 4 cm^2^ of the awning cloth were used. The analysis of PFOSA was possible in the awning cloth (in contrast to the matrix outdoors materials).

## Instrumental analysis

### HPLC parameters

Instrument: e.g. UPLC Acquity, Waters
Analytical column: 150 x 2 mm BEH C18, 1.7 µm, Waters
Flow: 0.25 mL/min
Injection volume: 20 µL

Table 5: Used chromatographic gradient:

| eluent A | 2 mM NH_4_Ac-methanol (95+5, v/v) | | |  |
| --- | --- | --- | --- | --- |
| eluent B | 2 mM NH_4_Ac in methanol | | |  |
| time (min) | A% | B% | flow (mL/min) | Gradient |
| 0 | 60 | 40 | 0.25 | Initial |
| 1 | 40 | 60 | 0.25 | 4 |
| 5 | 0 | 100 | 0.25 | 6 |
| 7 | 0 | 100 | 0.25 | 1 |
| 12 | 60 | 40 | 0.25 | 1 |

### MS-MS parameters

Instrument: e.g. TQD (Tandem Quadrupol Detector), Waters
Ionisation mode: electrospray negative (ES-)
Monitoring mode: MRM
Data system: Micromass Masslynx Software 4.0
Quantification: Internal standard quantification

Table 6: Mass transitions of target compounds and internal standards (IS)

| **Time, min** | **Compound Name** | **Parent Ion (m/z)** | **Daughter Ion (m/z)** | **Dwell (s)** | **cone volt. (V)** | **collision energy (eV)** |
| --- | --- | --- | --- | --- | --- | --- |
| 0 - 3.7 | PFBA | 212.83 | 168.7 | 0.05 | 20 | 11 |
|  | MPFBA (IS) | 216.9 | 172 | 0.05 | 19 | 10 |
|  | PFPA | 262.9 | 218.8 | 0.05 | 21 | 11 |
|  | PFBS | 298.9 | 79.7 | 0.05 | 47 | 35 |
|  | PFBS | 298.9 | 98.7 | 0.05 | 47 | 31 |
|  | PFHxA | 312.8 | 118.8 | 0.02 | 15 | 20 |
|  | PFHxA | 312.8 | 268.9 | 0.02 | 15 | 9 |
|  | MPFHxA (IS) | 314.9 | 269.8 | 0.02 | 15 | 9 |
| 3.7 - 4.65 | PFHpA | 362.9 | 168.8 | 0.02 | 15 | 15 |
|  | PFHpA | 362.9 | 318.8 | 0.02 | 15 | 9 |
|  | PFHxS | 398.9 | 79.7 | 0.02 | 60 | 33 |
|  | PFHxS | 398.9 | 98.7 | 0.02 | 60 | 37 |
|  | MPFHxS (IS) | 402.9 | 102.8 | 0.02 | 55 | 33 |
|  | PFOA | 412.9 | 168.8 | 0.02 | 17 | 21 |
|  | PFOA | 412.9 | 368.8 | 0.02 | 17 | 9 |
|  | MPFOA (IS) | 417.05 | 371.8 | 0.02 | 17 | 9 |
|  | PFHpS | 448.9 | 79.7 | 0.02 | 60 | 45 |
|  | PFHpS | 448.9 | 98.7 | 0.02 | 60 | 40 |
| 4.65 - 5.7 | PFNA | 462.9 | 168.8 | 0.02 | 20 | 23 |
|  | PFNA | 462.9 | 418.7 | 0.02 | 20 | 10 |
|  | MPFNA (IS) | 467.9 | 422.8 | 0.015 | 19 | 11 |
|  | PFOSA | 497.9 | 77.8 | 0.02 | 50 | 35 |
|  | PFOSA | 497.9 | 168.8 | 0.02 | 50 | 23 |
|  | PFOS | 498.9 | 79.8 | 0.02 | 60 | 41 |
|  | PFOS | 498.9 | 98.8 | 0.02 | 60 | 36 |
|  | MPFOS (IS) | 502.9 | 98.8 | 0.015 | 60 | 41 |
|  | PFDA | 512.9 | 268.8 | 0.02 | 19 | 17 |
|  | PFDA | 512.9 | 468.8 | 0.02 | 19 | 13 |
|  | MPFDA (IS) | 514.9 | 469.8 | 0.015 | 19 | 11 |
|  | PFUnA | 562.9 | 268.8 | 0.02 | 17 | 17 |
|  | PFUnA | 562.9 | 518.8 | 0.02 | 17 | 13 |
|  | MPFUnA (IS) | 564.9 | 519.9 | 0.015 | 17 | 9 |
|  | PFDS | 598.92 | 79.7 | 0.02 | 75 | 50 |
|  | PFDS | 598.92 | 98.7 | 0.02 | 75 | 41 |
| 5.7 -10 | PFDoA | 612.92 | 168.8 | 0.02 | 17 | 23 |
|  | PFDoA | 612.92 | 568.7 | 0.02 | 17 | 13 |
|  | MPFDoA (IS) | 614.9 | 569.8 | 0.02 | 17 | 13 |
|  | PFTrA | 662.92 | 168.8 | 0.02 | 23 | 27 |
|  | PFTrA | 662.92 | 618.7 | 0.02 | 23 | 13 |
|  | PFTeA | 712.92 | 168.8 | 0.02 | 33 | 33 |
|  | PFTeA | 712.92 | 668.7 | 0.02 | 33 | 11 |

## Identification

Identification is made by MS-MS and recording of the molecular ion and a minimum of one fragment ion. The analytes are identified by comparison of the MRM mass transitions (multiple reaction monitoring) and the retention times of standards and samples.

## Evaluation

Quantification is made by internal standardization using the mass labeled internal standards shown (IS) in Table 1. The IS are used for the quantification of the analytes as shown in Table 7.

Table 7: Quantification of analytes by internal standards

| **Mass labeled internal standard** | **Analyte(s)** |
| --- | --- |
| MPFBA | PFBA, PFPA |
| MPFHxA | PFHxA, PFHpA |
| MPFOA | PFOA |
| MPFNA | PFNA |
| MPFDA | PFDA |
| MPFUnA | PFUnA |
| MPFDoA | PFDoA, PFTrA, PFTeA |
| MPFHxS | PFBS, PFHxS, PFHpS |
| MPFOS | PFOS, PFDS, PFOSA |

Calibration is performed using a minimum of 6 calibration solutions. Calibration functions for every analyte are calculated with the concentration (x-axis) and the response (= area_analyte_ * (conc_IS_ /area_IS_)) (y-axis). Data acquisition and calculations are carried by the software Masslynx 4.X (Waters).

For the evaluation 2 mass transitions (if possible) are used for every analyte.

# Supplemental S2: Analytical methods for the determination of fluorotelomer alcohols (FTOHs) in air samples, impregnating sprays and solid samples

## Scope

The following analytical method describes the quantitative determination of 4 fluorotelomer alcohols und PFC precursors (see Table 8) in consumer products and air samples. Identification and quantification is carried out by the use of native standards and 4 deuterated and ^13^C-labelled internal standards (IS).

All the compounds mentioned in Table 8 were measured in this study, but due to method validation reasons only the 4 fluorotelomer alcohols 4:2, 6:2, 8:2 and 10:2 FTOH are reported.

Table 8: Fluorotelomer alcohols, perfluorosulfonamides and perfluorosulfonamido ethanols

| **Compounds** | | **Chemical formula** | **Structural formula** |
| --- | --- | --- | --- |
| N-MeFOSA | N-methylperfluoro-1-octanesulfonamide (CAS: 31506-32-8) | C_9_H_4_F_17_NO_2_S |  |
| N,N-Me_2_FOSA | N,N-dimethylperfluoro-1-octanesulfonamide (CAS: n.a.) | C_10_H_6_F_17_NO_2_S |  |
| N-EtFOSA | N-ethylperfluoro-1-octanesulfonamide (CAS: 4151-50-2) | C_10_H_6_F_17_NO_2_S |  |
| N-MeFOSE | 2-(N-methylperfluoro-1-octanesulfonamido)ethanol (CAS: 24448-09-7) | C_11_H_8_F_17_NO_3_S |  |
| N-EtFOSE | 2-(N-ethylperfluoro-1-octanesulfonamido)ethanol (CAS: 1691-99-2) | C_12_H_10_F_17_NO_3_S |  |
| FBET 4:2 FTOH | 2-Perfluorobutyl ethanol (4:2-telomeralcohol) (CAS: 2043-47-2) | C_6_H_5_F_9_O |  |
| FHET 6:2 FTOH | 2-Perfluorohexyl ethanol (6:2-telomeralcohol) (CAS: 647-42-7) | C_8_H_5_F_13_O |  |
| FOET 8:2 FTOH | 2-Perfluorooctyl ethanol (8:2-telomeralcohol) (CAS: 678-39-7) | C_10_H_5_F_17_O |  |
| FDET 10:2 FTOH | 2-Perfluorodecyl ethanol (10:2-telomeralcohol) (CAS: 865-86-1) | C_12_H_5_F_21_O |  |
| MFBET 4:2 FTOH | 2-Perfluorobutyl [1,1,2,2-^2^H_4_]-ethanol (4:2-telomeralcohol) (CAS: n.a.) | C_6_D_4_HF_9_O |  |
| MFHET 6:2 FTOH | 2-Perfluorohexyl [1,1-^2^H_2_]-[1,2-^13^C_2_]ethanol (6:2-telomeralcohol) (CAS: n.a.) | ^13^C_2_^12^C_6_D_2_H_3_F_13_O |  |
| MFOET 8:2 FTOH | 2-Perfluorooctyl [1,1-^2^H_2_]-[1,2-^13^C_2_]ethanol (8:2-telomeralcohol) (CAS: n.a.) | ^13^C_2_^12^C_8_D_2_H_3_F_17_O |  |
| MFDET 10:2 FTOH | 2-Perfluorodecyl [1,1-^2^H_2_]-[1,2-^13^C_2_]ethanol (10:2-telomeralcohol) (CAS: n.a.) | ^13^C_2_^12^C_8_D_2_H_3_F_21_O |  |

## Method summary

The analytes given in Table 8 were transferred into dichloromethane or hexane. Subsequent quantitative determination was performed by gas chromatography with mass spectrometric detection (GC-MS) after positive chemical ionisation.

## Equipment

- Glass equipment (pasteur pipettes, GC vials, etc.)
- vortexer, e.g. IKA Vibrax VXA
- nitrogen evaporator
- analytical balance
- drying cabinet
- refrigerator for reference standards
- refrigerator for samples
- GC-MS system, e.g. Finnigan MAT TSQ 7000

## Materials

### Reagents

Table 9: The following chemicals or reagents are examples, other brands of corresponding quality can be used.

| hexane | e.g. Merck, zur Analyse |
| --- | --- |
| acetone | e.g. Merck, zur Analyse |
| dichlormethane | e.g. Merck, zur Analyse |
| ethylacetate | e.g. Merck, zur Analyse |

### Solutions

Certified solutions

Certified solutions of the analytes with concentrations in the range of 2 µg/mL anion are used (e.g. Wellington Laboratories).

Internal standard solution

100 µL of each certified internal standard (50 µg/mL) are diluted with 100 µL hexane. The concentration is 10 µg/mL per standard. Every sample is fortified with 5 µL of this standard.

Dilutions

The solutions of the analytes are diluted with hexane to concentrations of 1 to 100 ng/mL. The calibration standards were prepared by adding 10 µL of the internal standard solution to each concentration level. The concentration of the internal standard is 100 ng/mL per standard.

Control standard solutions

Two control standard solutions with concentrations in the lower and upper part of the calibration range are used. The control standard solutions are prepared independently from the calibration solutions.

Injection standards

A solution of native N,N-Me_2_FOSA (50 ng/mL) is used as injection standard to check the recovery of the internal standards. It is checked before use, that the samples do not contain N,N-Me_2_FOSA.

## Performance

### Sample preparation

The PFCs analysed represent the amounts of the compounds which are extractable by the used methods.

- - 1. Air samples

The air volumes to be sampled are set to 5 – 50 m³ using a maximal sampling time of 48 hours (pump MZ C2 Synchor). These values resulted from the expected FTOH concentrations and the sampling outside of working hour.

Table 10 shows the materials tested as possible adsorber media for FTOHs. They are based on a polystyren-divinylbenzen copolymer (PVDB).

Table 10: Adsorber materials tested for the air sampling of FTOHs

| **Adsorber** | **Mass in cartridge [mg]** | **Volume [mL]** | **Article number** | **Manufacturer** |
| --- | --- | --- | --- | --- |
| OASIS WAX | 150 | 6 | 186002493 | Waters |
| ISOLUTE ENV+ | 200 | 6 | 915-0020-L | Separtis |
| Strata-X | 500 | 6 | 8B-S100-HCH-TN | Phenomenex |
| Supelclean ENVI-Carb | 500 | 6 | 57242 | Sigma-Aldrich |
| Atoll Columns Xtream Capacity | 500 | 6 | XC-70-9313 | Interchim |
| Strata Phenyl | 1000 | 6 | 8B-S006-JCH | Phenomenex |
| HyperSep Ratain PEP | 1000 | 25 | 60107-215 | Thermo Scienfitic |
| ISOLTUE ENV+ | 1000 | 25 | 915-0100-E | Biotage |

The FTOH elution from the adsorber cartridges was carried out following Jahnke et al., 2007a and 2007b. The procedure was optimized by the exchange of the elution solvent methanol by dichloromethane and addition of ethylacetate as keeper as evaporation of methanol eluates resulted in increased losses of analytes (recoveries of internal standards dropped below 50% in repeated trials).

The optimal sorption material was selected considering the recoveries and the blank values of breakthrough und reproducibility tests. The recovery was the main criterion for the selection.

The adsorbers OASIS WAX (150 mg) and ISOLUTE ENV+ (2x200 mg) exhibited the highest percentage recoveries. The adsorber ISOLUTE ENV+ (1000 mg) performed well also with a mean recovery of about 71 %. The recoveries of the other adsorption materials are clearly lower (e.g. < 50 %) or show breakdowns for the different analytes. Therefore they were not used as sorption materials in the air sampling of FTOHs.

The materials OASIS WAX (150 mg) and ISOLUTE ENV+ (2x200 mg) were not used because of their low bed mass in the available sorption cartridges. Therefore the adsorber material ISOLTUE ENV+ (1000 mg) was finally used.

- - 1. Emissions of consumer products

There were financial restrictions for the purchase or collection of sufficient amounts of some sample materials (especially clothing/textiles) for conventional analyses which require destructive sample preparations. For such samples the determination of the FTOH emissions was carried out.

The emissions were determined by placing the product into a 10 L desiccator equipped with an air in- and outlet. Through the outlet air was sucked over an SPE adsorber tube using a MZ C2 Synchor pump. The pressure in the desiccator was balanced via the air inlet. The emissions of the consumer products were determined over a time period of 3 h at 25 °C. The air volume sampled was 3 m³ and the air exchange rate was 100. Blank experiments were performed with an empty desiccator and the same set of parameters (3 h, 25 °C, 3 m³). FTOH levels in the blank experiment were lower than in the lowest emission sample (factors of 2; 25 and 20 for 6:2, 8:2 and 10:2 FTOH).

The processing of the SPE sampling tubes was identical to the processing of the air samples.

- - 1. Liquid samples

Liquid samples containing potentially FTOHs like impregnating sprays were transferred into glass vials. After dilution with acetone (1:10000) 5 µL of the internal standard solution was added. Recoveries tests were not employed as the samples were analysed directly by GC-MS without further processing steps.

- - 1. Solid samples

Solid samples were doted by the internal standards (^13^C-labelled) and extracted with an apolar solvent (hexane) in an ultrasonic bath for 15 min. Then extracts were centrifuged if needed and the solvent evaporated. If necessary the further silica cleanup steps following the procedure of Szostek et al. (2004) were carried out. The silica columns were conditioned with 2 mL of acetone and 2 mL of hexane. After addition of the extract the column was washed with 6 mL of hexane. The elution of the FTOHs was carried out using 1 mL of isopropanol. For the determination of the recovery the eluate was fortified with 4 µL injection standard.

## Instrumental parameters

### GC parameters (example)

Instrument: HP 5890 Series II
Analytical column: Phenomenex ZB624 60 m x 0.25 mm x 1.4 µm
Pressure: 23 psi
Injection volume: 2 µL

Temperature program: 60 °C (2 min) 🡪 20K/min, 260 °C (10 min)

### MS-MS parameters (example)

Instrument: e.g. Finnigan MAT TSQ7000 Tripelquadrupol
Ionisation mode: PCI with methane 5.5 as reaction gas, Linde
Filament emission: 300 µA
Electron energy: -50 eV
Source temperature: 180 °C
Monitoring mode: SIM
Data system: XCalibur
Quantification: Internal standard quantification

Table 11: FTOH molecular and fragment ions used

| **Compound** | **molecular and fragment ions used (fat: target m/z)** |
| --- | --- |
| 4:2 FTOH | 227, **265** |
| D_4_-4:2 FTOH | 231, **269** |
| 6:2 FTOH | 327, **365** |
| ^13^C_2_, D_2_ -6:2-FTOH | 331, **369** |
| 8:2 FTOH | 427, **465** |
| ^13^C_2_, D_2_ -8:2-FTOH | 431, **469** |
| 10:2 FTOH | 527, **565** |
| ^13^C_2_, D_2_ -10:2-FTOH | 531, **569** |
| N,N-Me_2_FOSA | **528** |
| N-MeFOSA | **514** |
| N-EtFOSA | **528** |
| N-MeFOSE | 540, **558** |
| N-EtFOSE | 554, **572** |

Table 12: FTOH limits of quantification (LOQ)

| **Product Group** | **Limit of Quantification (LOQ)** | |
| --- | --- | --- |
|  | **[µg/kg]** | **[µg/m^2^]** |
| Cleaning agents | 20000 | - |
| Carpets | - | 0.3 |
| Impregnating sprays | 5000 or 20000 depending on sample | - |
| Outdoor materials | - | 0.8 |
| Emissions of Outdoor materials | - | 0.4 ng/m^3^ |
| Mixed paper samples | 1.0 | - |

## Data acquisition

Data acquisition is carried by the software XCalibur (Thermo).

## Identification

Identification is made by MS-MS and recording of the molecular ion and a minimum of one fragment ion. The analytes are identified by comparison of the MRM mass transitions (multiple reaction monitoring) and the retention times of standards and samples.

## Evaluation

Quantification is made by internal standard method. Calibration is performed using 5 calibration solutions. Calibration functions for every analyte are calculated with the concentration (x-axis) and the response (= area_analyte_ * (conc_IS_ /area_IS_)) (y-axis). Calculations are performed using the XCalibur software.

For the evaluation 2 mass transitions (if possible) are used for every analyte.

## Control standards and blanks

Two control standard solutions with concentrations in the lower and upper part of the calibration range are measured at the beginning and the end of every analytical series and after about 20 samples.

In every analytical series a blank value is measured. In these blanks, the extraction or dilution solvents were applied without any sample contact and further treated as sample extracts and diluents.

Supplemental S3:

Table 13: PFAA contents of stored and recent paper based food contact materials

|  | **stock samples (before 2010)** | | | **recent samples (after 2010)** | | |
| --- | --- | --- | --- | --- | --- | --- |
|  | **min** | **max** | **median** | **min** | **max** | **median** |
| **PFBA** | 1.8 | 9.9 | 6.5 | -:- | 2.5 | -:- |
| **PFBS** | -:- | -:- | -:- | -:- | -:- | -:- |
| **PFDA** | 198.5 | 489.4 | 258.7 | -:- | 5.5 | -:- |
| **PFDoA** | 73.3 | 244.4 | 119.4 | -:- | 4.1 | -:- |
| **PFDS** | -:- | 1.7 | -:- | -:- | -:- | -:- |
| **PFHpA** | 42.0 | 379.3 | 106.3 | -:- | 0.6 | -:- |
| **PFHpS** | -:- | 0.8 | -:- | -:- | -:- | -:- |
| **PFHxA** | 38.8 | 182.8 | 64.0 | -:- | 2.5 | -:- |
| **PFHxS** | -:- | 0.6 | -:- | -:- | 0.6 | -:- |
| **PFNA** | 68.5 | 478.2 | 284.9 | -:- | 1.0 | -:- |
| **PFOA** | 219.4 | 658.1 | 363.1 | -:- | 13.5 | -:- |
| **PFOS** | 0.6 | 8.8 | 2.5 | -:- | 23.6 | 0.6 |
| **PFPA** | -:- | -:- | -:- | -:- | -:- | -:- |
| **PFTeA** | 11.6 | 33.3 | 23.6 | -:- | 18.0 | 1.0 |
| **PFTrA** | 3.5 | 22.1 | 9.7 | -:- | 1.5 | -:- |
| **PFUnD** | 5.2 | 36.6 | 17.8 | -:- | 0.6 | -:- |

Results are given as µg/kg. Stored samples: n = 3, recent samples: n = 12. -:- < Limit of Quantification (< 0.5 µg/kg)
